# Infectious Diseases Consultations as Markers of Hospital Workflow and Care Complexity

**DOI:** 10.3390/healthcare14131817

**Published:** 2026-06-23

**Authors:** Emel Gürcüoğlu

**Affiliations:** Department of Infectious Diseases and Clinical Microbiology, Bursa City Hospital, Bursa 16250, Türkiye; emel.gurcuoglu@saglik.gov.tr

**Keywords:** infectious disease consultation, COVID-19, intensive care, hospital organisation, mortality, artificial intelligence, clinical decision support

## Abstract

**Background/Objectives**: This preliminary, single-centre study evaluated infectious diseases consultation (IDC) patterns as indicators of hospital workflow and care complexity, aiming to characterise routinely available variables that may inform future organisational research and EHR-based clinical decision support development. **Methods:** In this retrospective study, 39,275 IDC requests from 16,430 patients were analysed using hospital information management system records. Paediatric patients and specialised immunosuppressed patient units were excluded. Request volumes, diagnostic categories, consultation purposes, and factors associated with in-hospital mortality were evaluated. Multivariable logistic regression models were constructed separately for two hospital blocks. **Results:** A total of 39,275 IDC records for 16,430 unique patients were reviewed. Mean consultation access time was 82.2 ± 64.3 min. Requests originated from surgical clinics (43.8%), followed by intensive care units (37.6%) and medical/internal clinics (18.6%). Pneumonia was the most common indication (30.5%), followed by unspecified infections (25.4%) and skin/soft tissue infections (17.2%). Consultation objectives included treatment, diagnostic assessment, and clinical guidance as non-mutually exclusive components. Significant block-level differences were observed in consultation timing, ICU-related consultation, diagnostic profiles, consultation purposes, and mortality. Age and ICU-related consultation were independently associated with mortality in both blocks, whereas consultation access time and COVID-19 diagnosis showed block-specific associations. **Conclusions:** IDC patterns may reflect not only diagnostic demand but also case severity, ICU-related care, consultation timing, and hospital location. As a preliminary single-centre study, these hypothesis-generating findings highlight the importance of integrating clinical, organisational, and contextual variables in future prospective, multi-centre studies aimed at developing EHR-based decision-support models. External validation, incorporation of comorbidity indices and microbiological data, and assessment of explainability are required before clinical implementation.

## 1. Introduction

During the COVID-19 pandemic, hospitals faced marked changes in patient flow, infection prevention practices, and antimicrobial use. These changes also affected the way infectious diseases consultations were requested and delivered. In centres with limited access to infectious diseases specialists, delayed consultation may complicate the management of healthcare-associated infections and antimicrobial-resistant pathogens [1,2,3]. Therefore, consultation processes need to be evaluated not only as a clinical service, but also as part of hospital workflow and infection management.

Infectious diseases specialists are often asked to support complex decisions, including antimicrobial selection, treatment modification, source control, interpretation of microbiological results, and assessment of clinical deterioration. When consultation demand increases, electronic health record-based tools may help identify high-risk patients and prioritise consultation requests [4,5]. However, such tools are useful only if their outputs are understandable and clinically credible. Prediction models that provide results without showing which clinical variables contributed to the decision may be difficult for physicians to trust or use in daily practice [5].

For this reason, before developing decision-support models, it is necessary to define which routinely available consultation variables reflect clinical risk and hospital workflow. Consultation timing, requesting unit, patient location, diagnostic category, and mortality may provide a practical basis for this purpose. In this study, we evaluated infectious diseases consultation patterns during and after the COVID-19 pandemic in a large tertiary hospital complex, with a focus on consultation timing, requesting units, diagnostic categories, and mortality-associated factors.

Clinical decision support systems (DSS) have been applied in various clinical domains, including sepsis recognition and severity prediction [6,7,8]; however, their use in infectious diseases consultation remains limited [8,9]. Most existing models rely on isolated clinical variables without incorporating organisational factors such as patient placement, unit type, or consultation timing, which may substantially influence outcomes [7,9]. For DSS to be clinically credible, the underlying variables must first be defined and validated using real-world hospital data [10].

The present study was therefore designed as a preliminary, single-centre investigation with two primary objectives: (1) to characterise IDC patterns across two structurally distinct hospital blocks during and after the COVID-19 pandemic, and (2) to identify consultation-level and patient-level variables associated with in-hospital mortality, with the aim of generating hypotheses and providing a local data source for future prospective and multi-centre research. We hypothesised that block-level organisational differences, ICU-related consultation burden, and consultation timing would be associated with differential mortality outcomes without restricting the analysis to a specific indication group.

## 2. Materials and Methods

### 2.1. Study Design

#### Study Setting

This was a retrospective observational evaluation of infectious diseases consultation services conducted between 2021 and 2025. The study was carried out in a large city hospital complex with a total capacity of 1435 beds according to 2025 data. The hospital had 245 ICU beds, including 18 level-one, 75 level-two, and 152 level-three ICU beds. Paediatric patients, obstetric patients, patients from the physical therapy unit, and highly specialised immunosuppressed patient groups concentrated in units such as haemato-oncology and radiation oncology were excluded.

Two hospital blocks representing adult patient workload and different clinical care profiles were included in the analysis. Block A had 250 beds and 45 ICU beds and mainly represented an area where cardiopulmonary and medical adult patients were followed. Block B had 258 beds and 59 ICU beds and reflected a predominantly surgical and critical care adult patient flow (Supplementary Table S1). There was also a structural difference between the two blocks in terms of ICU organisation. ICU beds in Block B were largely under the primary management of the Anaesthesiology and Reanimation team, which may have created an admission pattern with a higher concentration of critically ill, perioperative, or multi-organ support patients. In contrast, ICU patients in Block A were mostly followed within the ICU services of the relevant cardiopulmonary or medical specialties, while the Anaesthesiology and Reanimation team provided consultation support for these patients. During the COVID-19 pandemic, both blocks maintained their main clinical patient profiles but also served as principal areas for adult COVID-19 cases and COVID-19 patients requiring ICU care. Therefore, these two blocks were included to evaluate adult care burden, ICU-related clinical complexity, organisational differences between blocks, and the additional COVID-19-related care burden during the pandemic.

The primary unit of analysis was the infectious diseases consultation request. This approach was chosen to evaluate the workload, request characteristics, and clinical sources of the consultation service. Repeated consultations for the same patient were considered part of the service burden and clinical follow-up complexity. Analyses related to mortality were performed at the individual patient level.

Age, sex, hospital block, consultation year, consultation access time, requesting clinical unit, ICU status, diagnostic category, purpose of consultation, COVID-19 diagnosis, and mortality were evaluated.

Consultation access time was defined as the time interval between the electronic consultation request timestamp and the response or completion timestamp recorded in the hospital information management system. This interval reflects an operational documentation metric rather than actual bedside specialist contact time.

At the consultation-request level, ICU status was defined as a request originating from an ICU. At the patient level, ICU status was defined as the presence of at least one ICU-origin consultation request during the study period.

Diagnostic categories were derived from consultation request free-text fields in the hospital information management system using a systematic keyword-based screening approach. Predefined keyword lists were developed for each diagnostic category (Supplementary Table S2). Each consultation record was algorithmically screened against these keyword lists. When clinical context required disambiguation between overlapping categories, the full text of the consultation request was reviewed. This semi-automated approach was applied uniformly across all 39,275 records to ensure consistency and reproducibility. These categories included pneumonia, urinary tract infection, skin and soft tissue infection, sepsis, abdominal infection, neurological focus, bone and joint infection, viral infection, fever/observation, and general or undefined infection.

Consultation requests were evaluated not only by keyword screening but also by considering the clinical context and stated reasons for the request. Based on this evaluation, consultation purposes were classified into three main groups: treatment-oriented, diagnostic-oriented, and clinical-oriented requests (Supplementary Table S2). Treatment-oriented requests were mainly related to initiation, modification, de-escalation, determination of duration, or post-discharge planning of antimicrobial therapy. Diagnostic-oriented requests included evaluation of possible infection, fever or elevated acute-phase reactants, clinical interpretation of culture positivity, and investigation of the infection focus. Clinical-oriented requests included consultations related to clinical deterioration, non-response to treatment, suspected sepsis or septic shock, ICU follow-up, postoperative complications, wound or soft tissue problems, source control, or the need for multidisciplinary decision-making. When a request included more than one reason, each clinical purpose was coded as a separate binary variable; therefore, the same consultation record could be included in more than one purpose category.

Since a single consultation request could include more than one diagnostic category or consultation purpose, these variables were coded, when appropriate, as non-mutually exclusive binary variables. Therefore, the percentages for diagnostic categories and consultation purposes were not expected to total 100%.

COVID-19 diagnosis was defined according to the presence of a COVID-19-related diagnosis code or documentation in the consultation record. Mortality was defined according to the discharge status recorded in the hospital information management system and represented in-hospital mortality during the relevant hospitalisation episode.

### 2.2. Statistical Analysis

Continuous variables were summarised as mean ± standard deviation (SD) or median with interquartile range (IQR), and categorical variables were presented as numbers and percentages. Normality of continuous variables was assessed using the Kolmogorov–Smirnov test; since normality was not met, non-parametric tests were applied. The Mann–Whitney U test and chi-square test were used for comparisons between blocks. All statistical analyses were performed using IBM SPSS Statistics, Version 26.0 (IBM Corp., Armonk, NY, USA). Consultation-burden and workload analyses (Table 1) were based on 39,275 consultation records, whereas patient-level comparisons (Table 2) and multivariable regression models (Table 3) were based on 16,430 unique patients, using one observation per patient. Consultation-level variables were collapsed to patient-level summary measures before model fitting; specifically, consultation access time at the patient level represents the mean of all consultation access times recorded for each patient during the study period. Separate multivariable logistic regression models were constructed for each block to identify variables associated with mortality. These models were fitted at the patient level (Block A: n = 7517; Block B: n = 8913); since each patient contributed a single observation derived from patient-level summary measures, within-patient clustering of repeated consultation requests was structurally addressed at the aggregation step rather than through explicit multilevel modelling. Age, ICU status, consultation access time, COVID-19 diagnosis, and consultation year were included in the models. Diagnostic category and consultation purpose variables were not included in the regression models due to their non-mutually exclusive coding structure; multicollinearity among these overlapping binary variables would have compromised model stability, and this exclusion was a deliberate methodological choice rather than an attempt to claim independence from diagnostic category. Age was scaled per 10-year increase, and consultation access time was scaled per 60 min increase. Consultation year was included as a continuous variable. Results were reported as odds ratios (ORs), 95% confidence intervals, and *p* values.

**Figure 1:**
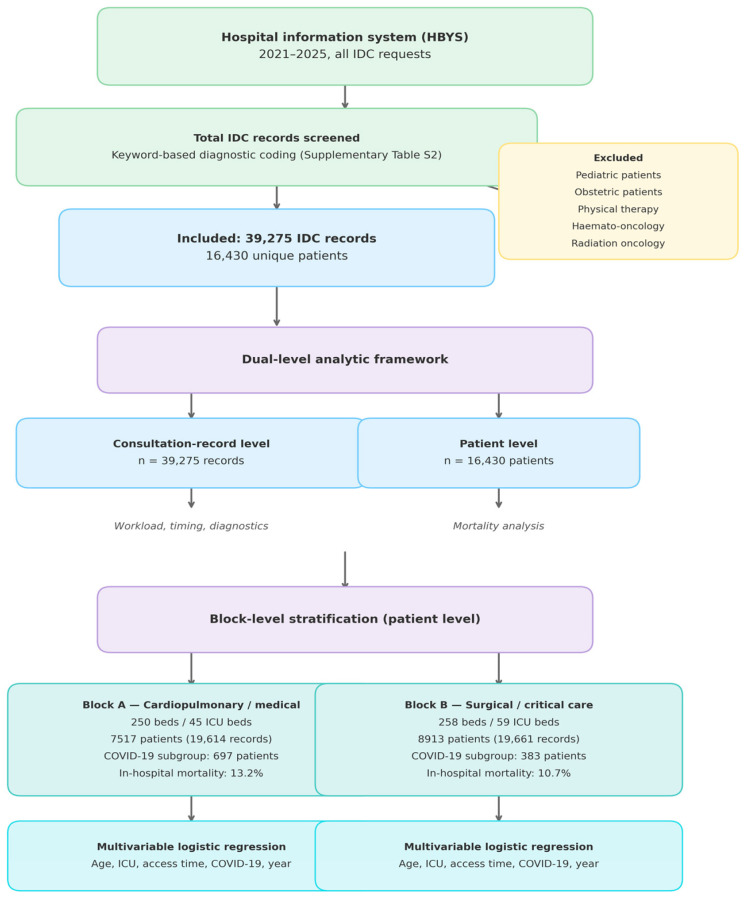
Study flow diagram showing patient and consultation record selection, exclusion criteria, dual-level analytic framework, and block-level stratification. IDC, infectious diseases consultation; ICU, intensive care unit; HBYS, hospital information management system.

The study design, exclusion criteria, dual-level analytic framework, and block-level stratification are summarised in Figure 1.

## 3. Results

A total of 39,275 infectious diseases consultation records belonging to 16,430 unique patients from both blocks were reviewed. Patient age ranged from 18 to 110 years. The mean consultation access time was 82.2 ± 64.3 min, and the median was 64 min (Q1–Q3: 30–121; range: 1–294). Consultation requests most frequently originated from surgical departments (43.8%; 17,189), followed by ICUs (37.6%; 14,761) and medical departments (18.6%; 7305) (Table 1).

The most common reason for consultation was pneumonia-related evaluation (11,977; 30.5%). This was followed by general or undefined infection requests (9990; 25.4%), skin and soft tissue infections (6741; 17.2%), urinary tract infections (5376; 13.7%), and sepsis (5143; 13.1%). Consultation purposes included treatment-related components in 59.5% of requests, diagnostic components in 60.9%, and clinical guidance components in 57.2%. Since diagnostic categories and consultation purposes were not mutually exclusive, the same record could be included in more than one category, and the percentages were not expected to total 100% (Table 1). Consultation access time was summarised at the consultation-record level in Table 1 and at the patient level in Table 2; therefore, summary values differ between these tables.

At the patient level, significant differences were observed between Block A and Block B in terms of consultation access time, age, sex, ICU status, requesting unit distribution, diagnostic categories, consultation purposes, and mortality (Table 2).

When patients with a COVID-19 diagnosis were evaluated separately, consultation records were found to be concentrated mainly in the 2021–2022 period. In Block A, 1326 consultation records were identified for 697 unique patients with COVID-19 diagnosis, whereas in Block B, 710 consultation records were identified for 383 unique patients. At the unique patient level, in-hospital mortality was 7.3% in Block A and 27.9% in Block B. Mortality was particularly high in Block B in 2021, when death occurred in 103 of 291 unique patients (Table 4 and Table 5).

The yearly distribution and outcomes of COVID-19-diagnosed patients are presented in Table 4 for Block A and Table 5 for Block B. Since annual patient numbers were low, particularly during the 2023–2025 period, mortality percentages for these years should be interpreted with caution (Table 4 and Table 5).

In multivariable logistic regression analysis, age was independently associated with mortality in both blocks. For each 10-year increase in age, the odds of mortality increased 1.27-fold in Block A (95% CI: 1.21–1.34; *p* < 0.001) and 1.25-fold in Block B (95% CI: 1.20–1.31; *p* < 0.001). ICU-origin consultation was also strongly associated with mortality in both blocks (Block A OR: 7.69, 95% CI: 6.52–9.07; *p* < 0.001; Block B OR: 30.43, 95% CI: 24.39–37.96; *p* < 0.001). Each 60 min increase in consultation access time was not significantly associated with mortality in Block A (OR: 1.01, 95% CI: 0.96–1.07; *p* < 0.001), whereas it was significantly associated with increased odds of mortality in Block B (OR: 1.09, 95% CI: 1.04–1.15; *p* < 0.001). COVID-19 diagnosis was associated with lower odds of mortality in Block A (OR: 0.51, 95% CI: 0.40–0.65; *p* < 0.001), but with higher odds of mortality in Block B (OR: 1.85, 95% CI: 1.44–2.38; *p* < 0.001). Consultation year was also independently associated with mortality in both blocks, with a stronger association in Block B (Block A OR: 1.09, 95% CI: 1.03–1.14; *p* = 0.002; Block B OR: 1.19, 95% CI: 1.13–1.26; *p* < 0.001) (Table 3).

In addition, the clinical units included in the study are presented in Supplementary Table S1, and the operational definitions and keywords used for consultation-purpose classification are provided in Supplementary Table S2.

## 4. Discussion

IDC should not be regarded only as a quantitative measure of workload, but also as an indicator of hospital-level clinical burden, care complexity, and workflow-related factors. As a preliminary, single-centre descriptive study, the present work was not designed to establish causal relationships or develop a predictive model, but rather to characterise routinely available consultation variables as a potential data source for future organisational studies and clinical decision-support development. Our experience during the pandemic suggests that consultation traffic may serve as a useful source of information on hospital functioning, particularly when interpreted together with capacity pressure, sudden increases in demand, and disruptions in referral and workflow processes [11,12].

In this context, the findings from Block B should be interpreted with caution. At the patient level, crude mortality was not higher in Block B than in Block A; on the contrary, mortality was 13.2% in Block A and 10.7% in Block B, as shown in Table 2. However, multivariable models showed that ICU-related consultation and longer consultation access time were more strongly associated with mortality in Block B. Within the limits of the retrospective design, this association should not be interpreted as a causal effect. Rather, it suggests that mortality and poor clinical course may not be explained solely by the patient’s baseline severity. The findings indicate that the requesting unit’s familiarity with the patient, the appropriateness of ongoing clinical management, and the timing of consultation may also contribute to outcomes. It should be noted that consultation access time in this study was derived from documentation timestamps in the hospital information system, and may reflect documentation lag, organisational complexity, or patient acuity rather than a direct measure of specialist delay. Any association between access time and mortality should therefore be interpreted with caution and should not be construed as causal. Future studies should examine whether year- and unit-specific access time distributions differ systematically, and whether adjusting for documentation practices alters the observed associations. Therefore, the emphasis should be placed less on the difference between blocks and more on the quality of consultation and the clinical control of the requesting team. The observed association between access time and mortality in Block B may also reflect residual confounding from unmeasured variables such as comorbidity burden and acute illness severity, which were not available for model adjustment. Accordingly, this finding should be regarded as a hypothesis-generating observation rather than evidence of a causal pathway involving consultation delay over the patient’s course.

The marked difference in sepsis-coded consultations between blocks may partly reflect differences in clinical documentation practices, ICU organisation, and the case-mix of surgical critical care services. This finding should therefore be interpreted as a signal of organisational and documentation-related heterogeneity rather than as a direct measure of true sepsis incidence across blocks. For example, a critically ill patient with pulmonary infection and organ dysfunction may be coded under pneumonia-related keywords in Block A (where cardiopulmonary specialties predominate) and under sepsis-related keywords in Block B (where Anaesthesiology and Reanimation teams use sepsis-oriented clinical terminology). The possibility that diagnostic coding categories were applied inconsistently across clinical units cannot be excluded; however, no formal interrater reliability assessment was performed in this study, which represents a methodological limitation. Future studies should incorporate a structured validation step for EHR-derived diagnostic categories.

This observation is conceptually consistent with studies reporting an association between early ID consultation and clinical outcomes in different patient groups. Madaline et al. showed that early ID consultation within the first 12 h reduced in-hospital mortality in patients with severe sepsis [13]. In a population-wide cohort study, Ong et al. also demonstrated that ID consultation was independently associated with reduced mortality among patients with Gram-negative bloodstream infections [14]. However, since the present study focused on all IDC requests rather than on a specific infection group, a direct causal comparison with these studies would not be appropriate.

In addition, the strong association observed between ICU-origin consultation and mortality in Block B (OR: 30.43) suggests that ID consultations should be interpreted not only in relation to individual disease severity, but also in relation to the care setting, ICU organisation, and hospital workflow. Capacity pressure, sudden pandemic-like increases in demand, ICU burden, and interruptions in referral and workflow processes should be considered as possible system-level contributors to this association [11,12]. The magnitude of this association (OR: 30.43) likely reflects the fact that ICU-origin consultation serves as a proxy for disease severity rather than an independent risk factor; patients referred from ICUs represent a pre-selected population with substantially higher baseline mortality risk.

Similarly, the opposite direction of the association between COVID-19 diagnosis and mortality in the two blocks suggests that mortality cannot be explained by the diagnostic label alone. This difference may reflect several interacting factors: (i) period-specific triage and admission policies that differed between pandemic waves; (ii) ICU organisational structure, with Block B ICUs primarily managed by Anaesthesiology and Reanimation, potentially concentrating higher-acuity COVID-19 patients; (iii) patient placement decisions driven by bed availability rather than clinical matching; and (iv) differences in clinical documentation and coding practices between blocks [15]. No causality should be inferred from this observational finding.

These findings suggest that risk assessment based only on classical clinical scores such as ESI or NEWS [16].may not fully capture the influence of organisational and contextual factors on patient outcomes. Although the present study did not develop or validate a predictive model, the observed associations suggest that future studies should consider not only patient-level clinical features but also care environment, consultation timing, and organisational variables [7,8,10]. External validation across multiple institutions is necessary before any decision-support application can be recommended for routine use.

### 4.1. Implications of the Findings and Future Research Directions

These preliminary findings have potential implications for hospital infection management and future research. IDC data derived from hospital information systems may serve as a routinely available data source for characterising clinical complexity beyond simple workload metrics. The observed differential associations between organisational variables and mortality across blocks underscore the importance of contextual and structural factors in clinical outcome analysis. Importantly, the present study was designed to provide a descriptive foundation and local data source for future organisational studies with similar institutional settings. The identified variables may contribute to the development of patient prioritisation frameworks and consultation triage tools; however, such applications require prospective validation, incorporation of comorbidity indices, organ failure scores, microbiological data, and antimicrobial timing, as well as external validation across multiple hospital settings before any model can be recommended for clinical use [7,8,9,10].

### 4.2. Study Limitations

This study has several limitations. First, the retrospective design precludes causal inference; associations between consultation variables and mortality may reflect pre-existing severity or selection effects. In addition, although consultation-level variables were aggregated to patient-level summary measures (one observation per patient), this aggregation approach may not fully capture the clinical complexity of patients with multiple consultations; future analyses using generalised estimating equations or mixed-effects models applied to consultation-level data would provide a more granular assessment. Second, the multivariable models did not include comorbidity burden, organ failure scores, infection source, microbiological data, or antimicrobial timing, which limits the clinical interpretability of the reported odds ratios. Third, the patient-level ICU definition (presence of at least one ICU-origin consultation) may introduce reverse causation bias, as ICU transfer may represent deterioration that preceded the consultation. Fourth, although diagnostic coding was based on systematic, predefined keyword screening rather than subjective manual classification, the keyword lists were developed by a single investigator and were not independently validated by a second reviewer; minor misclassification at the boundaries of overlapping categories cannot be excluded, and documentation practices may differ across blocks. Fifth, the analysis was conducted in a single hospital complex, and the exclusion of paediatric, obstetric, physical therapy, and highly specialised immunosuppressed patient groups limits the generalizability of the findings to adult, non-immunosuppressed hospital populations. Finally, consultation counts were not normalised to hospital activity (total admissions, bed-days, or ICU occupancy), which may affect interpretation of workload trends.

## 5. Conclusions

In conclusion, IDC patterns may reflect not only diagnostic demand but also care setting, ICU burden, consultation timing, and hospital organisation. These variables should be evaluated together in prospectively designed, multi-centre studies that also incorporate comorbidity indices, microbiological data, and antimicrobial timing. Before any consultation-derived variable is used in clinical risk stratification, prospective external validation across diverse hospital settings is required. These findings underscore that organisational metrics such as consultation timing, unit of origin, and patient placement require explicit assessment for transparency and clinical interpretability in any future EHR-based model.

## Figures and Tables

**Table 1:** Overall Infectious Diseases Consultation Burden.

Variable	Total
Total consultation records, n	39,275
Age range, years	18–110
Consultation access time, min, mean ± SD	82.2 ± 64.3
Consultation access time, min, median (Q1–Q3)	64 (30–121)
Consultation access time, min, range	1–294
Intensive care unit requests, n (%)	14,761 (37.6)
Medical department requests, n (%)	7305 (18.6)
Surgical department requests, n (%)	17,189 (43.8)
Pneumonia, n (%)	11,977 (30.5)
Urinary tract infection, n (%)	5376 (13.7)
Skin and soft tissue infection, n (%)	6741 (17.2)
Sepsis, n (%)	5143 (13.1)
Abdominal infection, n (%)	1199 (3.1)
Neurological focus, n (%)	232 (0.6)
Bone-joint infection, n (%)	3036 (7.7)
Viral infection, n (%)	2383 (6.1)
Fever/observation, n (%)	4386 (11.2)
General/undetermined, n (%)	9990 (25.4)
Treatment-oriented request, n (%)	23,359 (59.5)
Diagnostic-oriented request, n (%)	23,926 (60.9)
Clinically oriented request, n (%)	22,461 (57.2)

Note: Diagnostic categories and consultation-purpose categories were coded as non-mutually exclusive variables; therefore, percentages do not sum to 100%.

**Table 2:** Patient-Level Comparison Between Block A and Block B.

Variable	Total (N = 16,430)	Block A (n = 7517)	Block B (n = 8913)	*p*-Value
Consultation access time, min	95.8 ± 325.5; 65 (30–125)	73 (33–137)	60 (28–115)	<0.001
Age, years	65.4 ± 16.7; 68 (56–77)	71 (61–79)	66 (53–75)	<0.001
Female sex, n (%)	6654 (40.5)	2842 (37.8)	3812 (42.8)	<0.001
Male sex, n (%)	9776 (59.5)	4675 (62.2)	5101 (57.2)	<0.001
ICU-related consultation, n (%)	4741 (28.9)	2351 (31.3)	2390 (26.8)	<0.001
Surgical department request, n (%)	8421 (51.3)	1898 (25.2)	6523 (73.2)	<0.001
Pneumonia, n (%)	4752 (28.9)	3979 (52.9)	773 (8.7)	<0.001
Urinary tract infection, n (%)	2478 (15.1)	979 (13.0)	1499 (16.8)	<0.001
Skin and soft tissue infection, n (%)	2880 (17.5)	1484 (19.7)	1396 (15.7)	<0.001
Sepsis, n (%)	1800 (11.0)	11 (0.1)	1789 (20.1)	<0.001
Abdominal infection, n (%)	561 (3.4)	435 (5.8)	126 (1.4)	<0.001
Neurological focus, n (%)	75 (0.5)	62 (0.8)	13 (0.1)	<0.001
Bone-joint infection, n (%)	1361 (8.3)	1195 (15.9)	166 (1.9)	<0.001
Viral infection, n (%)	1324 (8.1)	863 (11.5)	461 (5.2)	<0.001
Fever/observation, n (%)	1908 (11.6)	1277 (17.0)	631 (7.1)	<0.001
General/undetermined, n (%)	4294 (26.1)	1110 (14.8)	3184 (35.7)	<0.001
Treatment-oriented request, n (%)	9942 (60.5)	4396 (58.5)	5546 (62.2)	<0.001
Diagnostic-oriented request, n (%)	9448 (57.5)	5387 (71.7)	4061 (45.6)	<0.001
Clinically oriented request, n (%)	8829 (53.7)	4796 (63.8)	4033 (45.2)	<0.001
In-hospital mortality, n (%)	1948 (11.9)	990 (13.2)	958 (10.7)	<0.001

Note: Continuous variables are presented as mean ± SD and median (Q1–Q3) in the total column and as median (Q1–Q3) in the group columns, according to the available source data. Categorical variables are presented as n (%). Between-block comparisons were performed using the Mann–Whitney U test for continuous variables and the chi-square test for categorical variables. All *p*-values are two-sided; results should be interpreted as descriptive unless otherwise specified. Given the large sample size, absolute differences between groups should be considered alongside *p*-values when assessing clinical relevance. Absolute differences (Block A minus Block B): consultation access time median difference +13 min; age median difference +5 years; female sex −5.0%; ICU-related consultation +4.5%; surgical department −48.0%; pneumonia +44.2%; UTI −3.8%; SSTI +4.0%; sepsis −20.0%; abdominal infection +4.4%; neurological focus +0.7%; bone-joint infection +14.0%; viral infection +6.3%; fever/observation +9.9%; general/undetermined −20.9%; treatment-oriented −3.7%; diagnostic-oriented +26.1%; clinically oriented +18.6%; in-hospital mortality +2.5%.

**Table 3:** Multivariable Logistic Regression Analysis of Factors Associated with In-Hospital Mortality.

Variable	Block A OR	95% CI	*p*-Value	Block B OR	95% CI	*p*-Value
Age (per 10-year increase)	1.27	1.21–1.34	<0.001	1.25	1.20–1.31	<0.001
At least one ICU-origin consultation	7.69	6.52–9.07	<0.001	30.43	24.39–37.96	<0.001
Consultation access time (per 60 min increase)	1.01	0.96–1.07	0.589	1.09	1.04–1.15	<0.001
COVID-19 diagnosis	0.51	0.40–0.65	<0.001	1.85	1.44–2.38	<0.001
Consultation year (per calendar year)	1.09	1.03–1.14	0.002	1.19	1.13–1.26	<0.001

Note. The outcome variable was in-hospital mortality, defined according to discharge status. ICU admission was defined as the presence of at least one consultation request originating from an intensive care unit. COVID-19 diagnosis was identified based on COVID-related text or codes in the diagnosis, consultation diagnosis, consultation request reason, or requesting unit fields. Age was scaled per 10-year increase, Consultation access time per 60 min increase, and consultation year was modelled as a continuous variable per calendar year. OR, odds ratio; CI, confidence interval; ICU, intensive care unit.

**Table 4:** Distribution and Outcomes of COVID-19-Diagnosed Cases in Block A.

Variable	Overall	2021	2022	2023	2024	2025
Consultation records, n	1326	910	331	19	59	7
Unique patients, n	697	502	159	9	24	3
Deaths, n	51	22	23	2	4	0
In-hospital mortality, %	7.3	4.4	14.5	22.2	16.7	0
Female sex, n (%)	290 (41.6)	—	—	—	—	—
Male sex, n (%)	407 (58.4)	—	—	—	—	—
Age, mean ± SD, years	66.8 ± 15.4	—	—	—	—	—
Age, median, years	68.1	—	—	—	—	—
Consultations per patient, median	1	—	—	—	—	—
Consultations per patient, range	1–11	—	—	—	—	—

**Table 5:** Distribution and Outcomes of COVID-19-Diagnosed Cases in Block B.

Variable	Overall	2021	2022	2023	2024	2025
Consultation records, n	710	493	187	7	19	4
Unique patients, n	383	291	78	3	10	1
Deaths, n	107	103	0	0	3	1
In-hospital mortality, %	27.9	35.4	0	0	30.0	100.0
Female sex, n (%)	169 (44.1)	—	—	—	—	—
Male sex, n (%)	214 (55.9)	—	—	—	—	—
Age, mean ± SD, years	65.4 ± 17.0	—	—	—	—	—
Age, median, years	67.4	—	—	—	—	—
Consultations per patient, median	1	—	—	—	—	—
Consultations per patient, range	1–15	—	—	—	—	—

Note: Mortality was calculated at the unique-patient level and reflects in-hospital mortality according to discharge status. Consultation records represent repeated consultation requests, whereas unique patients represent patient-level counts. Percentages for years with very small patient numbers, particularly 2023–2025, should be interpreted cautiously.

## Data Availability

The datasets generated and/or analysed during the current study are not publicly available due to institutional and privacy restrictions but may be available from the corresponding author upon reasonable request and with permission from the relevant institution.

## Supplementary Materials

The following supporting information can be downloaded at: https://www.mdpi.com/article/10.3390/healthcare14131817/s1, Table S1. Clinical Units Included in Block A and Block B. Table S2. Keywords and Phrases Used for Classification of Consultation Purpose.

## References

[1] Taie A., Gheorghe M., Amos J., Morton A., Gordon J., Jenkins N.C., Padgett T.E., Hollinghurst J., Taylor G. (2025). Antimicrobial Resistance Trends, Predictors, and Burden in England: A Retrospective Study Using the Clinical Practice Research Datalink from 2015 to 2021. Int. J. Antimicrob. Agents.

[2] Boccabella L., Palma E.G., Abenavoli L., Scarlata G.G.M., Boni M., Ianiro G., Santori P., Tack J.F., Scarpellini E. (2024). Post-Coronavirus Disease 2019 Pandemic Antimicrobial Resistance. Antibiotics.

[3] Zwerwer L.R., Luz C.F., Soudis D., Giudice N., Nijsten M.W., Glasner C., Renes M.H., Sinha B. (2024). Identifying the Need for Infection-Related Consultations in Intensive Care Patients Using Machine Learning Models. Sci. Rep..

[4] Abbara S., Crabol Y., de Bouillé J.G., Dinh A., Morquin D. (2025). Artificial Intelligence and Infectious Diseases: Scope and Perspectives. Infect. Dis. Now..

[5] Khalid L., Saleem K., Mushtaq S., Hussain I., Hussain Z., Hussain Z., Paracha R.Z., Khan A., Chang J., Fang Y. (2026). Global Prediction of Antimicrobial Resistance Trends Using Statistical and Machine Learning Models: Evaluating National Action Plan Policy Impacts through Interrupted Time Series Analysis. J. Glob. Antimicrob. Resist..

[6] Marcos M., Belhassen-García M., Sánchez-Puente A., Sampedro-Gomez J., Azibeiro R., Dorado-Díaz P.I., Marcano-Millán E., García-Vidal C., Moreiro-Barroso M.-T., Cubino-Bóveda N. (2021). Development of a Severity of Disease Score and Classification Model by Machine Learning for Hospitalized COVID-19 Patients. PLoS ONE.

[7] Sutton R.T., Pincock D., Baumgart D.C., Sadowski D.C., Fedorak R.N., Kroeker K.I. (2020). An Overview of Clinical Decision Support Systems: Benefits, Risks, and Strategies for Success. NPJ Digit. Med..

[8] Dahò M., Caci B. (2025). Clinical Decision Support Systems and Artificial Intelligence in Assessment and Rehabilitation: Opportunities and Challenges for Technology-Assisted Care. Healthcare.

[9] Almadani B., Kaisar H., Thoker I.R., Aliyu F. (2025). A Systematic Survey of Distributed Decision Support Systems in Healthcare. Systems.

[10] Dos Santos F.C., Snigurska U.A., Keenan G.M., Lucero R.J., Modave F. (2023). Clinical Decision Support Systems for Palliative Care Management: A Scoping Review. J. Pain Symptom Manag..

[11] Hadano Y., Matsumoto T. (2023). Non-Infectious Diseases in Infectious Disease Consultation: A Descriptive Study in a Tertiary Care Teaching Hospital. PLoS ONE.

[12] Hadano Y., Kakuma T., Matsumoto T., Ishibashi K., Isoda M., Yasunaga H. (2021). Reduction of 30-Day Death Rates from Staphylococcus Aureus Bacteremia by Mandatory Infectious Diseases Consultation: Comparative Study Interventions with and without an Infectious Disease Specialist. Int. J. Infect. Dis..

[13] Madaline T., Wadskier Montagne F., Eisenberg R., Mowrey W., Kaur J., Malik M., Gendlina I., Guo Y., White D., Pirofski L.A. (2019). Early Infectious Disease Consultation Is Associated with Lower Mortality in Patients with Severe Sepsis or Septic Shock Who Complete the 3-Hour Sepsis Treatment Bundle. Open Forum Infect. Dis..

[14] Ong S.W., Luo J., Fridman D.J., Lee S.M., Johnstone J., Schwartz K.L., Diong C., Patel S.N., MacFadden D.R., Langford B.J. (2024). Association between Infectious Diseases Consultation and Mortality in Hospitalized Patients with Gram-Negative Bloodstream Infection: A Retrospective Population-Wide Cohort Study. Clin. Infect. Dis..

[15] Pormohammad A., Ghorbani S., Khatami A., Razizadeh M.H., Alborzi E., Zarei M., Idrovo J.P., Turner R.J. (2021). Comparison of Influenza Type A and B with COVID-19: A Global Systematic Review and Meta-Analysis on Clinical, Laboratory and Radiographic Features. Rev. Med. Virol..

[16] Durr D., Niemi T., Despraz J., Tusgul S., Dami F., Akrour R., Carron P.N., Le Pogam M.A., Calandra T., Meylan S. (2022). National Early Warning Score (NEWS) Outperforms Quick Sepsis-Related Organ Failure (qSOFA) Score for Early Detection of Sepsis in the Emergency Department. Antibiotics.

