# Supplementary material for: Infectious Diseases Consultations as Markers of Hospital Workflow and Care Complexity
Source: Healthcare (Basel). 2026 Jun 23;14(13):1817. doi: 10.3390/healthcare14131817 (PMC13360685; doi:10.3390/healthcare14131817)
Supplement: Supplementary file 1 [file healthcare-14-01817-s001.zip › healthcare-4365275-supplementary.pdf]

Supplementary Table S1. Clinical Units Included in Block A and Block B

Block A	Block B
Chest Diseases	Urology
Cardiology	General Surgery
Otorhinolaryngology	Surgical Oncology
Cardiovascular Surgery	Gastrointestinal Surgery
Thoracic Surgery	Neurosurgery
Chest Diseases Intensive Care Unit	Orthopedics and Traumatology
Coronary Intensive Care Unit	Burn Ward
Prisoner Ward / General Surgery–Internal Medicine	Burn Intensive Care Unit
	Surgical Intensive Care Unit
	Anaesthesiology and Reanimation Intensive Care Unit
	General Intensive Care Unit
	Transplantation / Nephrology–General Surgery

Note. *Blocks A and B represent the adult inpatient care areas included in the analysis. Paediatric, obstetric, physical therapy, haemato-oncology, radiation oncology, and other highly specialized immunosuppressed patient units were excluded from the study.*

Supplementary Table S2. Keywords and Phrases Used for Classification of Consultation Purpose

Consultation purpose category	Operational definition	Keywords and phrases used for classification
Treatment-oriented consultations	Consultations primarily requested for the initiation, adjustment, modification, de-escalation, continuation, duration planning, intravenous-to-oral switch, or discharge planning of antimicrobial therapy in patients with known or suspected infection.	Antibiotic initiation; antibiotic prescription; antibiotic adjustment; antibiotic recommendation; antibiotic modification; antibiotic revision; empirical treatment; targeted treatment; culture-guided treatment; treatment adjustment; treatment change; treatment duration; treatment continuation; treatment discontinuation; de-escalation; escalation; intravenous treatment; oral treatment; oral switch; discharge treatment; discharge antibiotic; prescription recommendation; prophylaxis; preoperative prophylaxis; postoperative antibiotic; antifungal treatment; antiviral treatment.
Diagnostic-oriented consultations	Consultations primarily requested to determine whether infection was present, to clarify the possible infectious focus or causative agent, or to interpret microbiological and inflammatory findings.	Evaluation for infection; possible infection; infectious focus; focus investigation; focus clarification; fever; fever of unknown origin; elevated acute-phase reactants; elevated C-reactive protein; elevated procalcitonin; leukocytosis; elevated erythrocyte sedimentation rate; culture growth; blood culture growth; urine culture growth; wound culture growth; sputum culture growth; culture positivity; contamination; colonization; clinical significance of growth; pathogen assessment; exclusion of infection; further diagnostic work-up; additional laboratory testing; imaging recommendation.
Clinically oriented consultations	Consultations requested in the context of clinical deterioration, lack of response to treatment, critical illness, postoperative complications, wound or soft-tissue problems, source-control issues, or the need for multidisciplinary decision-making.	Clinical deterioration; poor general condition; lack of response; treatment failure; persistent fever; newly developed fever; sepsis; septic shock; hypotension; intensive care; intubated patient; mechanical ventilation; postoperative complication; complication; wound problem; wound infection; soft-tissue problem; abscess; drainage; source control; surgical evaluation; multidisciplinary evaluation; respiratory deterioration; worsening pneumonia; deterioration during follow-up; no clinical response; fatal course; critically ill patient.
